# Characterization of pediatric Philadelphia-negative B-cell precursor acute lymphoblastic leukemia with kinase fusions in Japan

**DOI:** 10.1038/bcj.2016.28

**Published:** 2016-05-13

**Authors:** T Imamura, N Kiyokawa, M Kato, C Imai, Y Okamoto, M Yano, K Ohki, Y Yamashita, Y Kodama, A Saito, M Mori, S Ishimaru, T Deguchi, Y Hashii, Y Shimomura, T Hori, K Kato, H Goto, C Ogawa, K Koh, T Taki, A Manabe, A Sato, A Kikuta, S Adachi, K Horibe, A Ohara, A Watanabe, Y Kawano, E Ishii, H Shimada

**Affiliations:** 1 Department of Pediatrics, Kyoto Prefectural University of Medicine, Graduate School of Medical Science, Kyoto, Japan; 2 Department of Pediatric Hematology and Oncology Research, National Research Institute for Child Health and Development, Tokyo, Japan; 3 Department of Pediatrics, The University of Tokyo, Tokyo, Japan; 4 Division of Pediatrics, Department of Homeostatic Regulation and Development, Niigata University Graduate School of Medical and Dental Sciences, Niigata, Japan; 5 Department of Pediatrics, Graduate School of Medical and Dental Sciences, Kagoshima University, Kagoshima, Japan; 6 Department of Hematology/Oncology, Gunma Children's Medical Center, Shibukawa, Japan; 7 National Hospital Organization Nagoya Medical Center, Clinical Research Center, Nagoya, Japan; 8 Department of Hematology and Oncology, Hyogo Prefectural Children's Hospital, Kobe, Japan; 9 Department of Hematology/Oncology, Saitama Children's Medical Center, Saitama, Japan; 10 Department of Hematology/Oncology, Tokyo Metropolitan Children's Medical Center, Tokyo, Japan; 11 Department of Pediatrics, Mie University, Tsu, Japan; 12 Department of Pediatrics, Osaka University, Osaka, Japan; 13 Department of Pediatrics, Aichi Medical University School of Medicine, Nagakute, Japan; 14 Division of Pediatric Hematology/Oncology, Ibaraki Children's Hospital, Mito, Japan; 15 Division of Hemato-Oncology and Regenerative Medicine, Kanagawa Children's Medical Center, Yokohama, Japan; 16 Department of Pediatric Oncology, National Cancer Center Hospital, Tokyo, Japan; 17 Department of Molecular Diagnostics and Therapeutics, Kyoto Prefectural University of Medicine, Kyoto, Japan; 18 Department of Pediatrics, St Luke's International Hospital, Tokyo, Japan; 19 Department of Hematology and Oncology, Miyagi Children's Hospital, Sendai, Japan; 20 Department of Pediatrics, Fukushima Medical School, Fukushima, Japan; 21 Department of Human Health Sciences, Graduate School of Medicine, Kyoto University, Kyoto, Japan; 22 Clinical Research Center, National Hospital Organization Nagoya Medical Center, Nagoya, Japan; 23 Department of Pediatrics, Toho University, Tokyo, Japan; 24 Department of Pediatrics, Nakadori General Hospital, Akita, Japan; 25 Department of Pediatrics, Ehime University Graduate School of Medicine, Toon, Japan; 26 Department of Pediatrics, School of Medicine, Keio University School of Medicine, Tokyo, Japan

## Abstract

Recent studies revealed that a substantial proportion of patients with high-risk B-cell precursor acute lymphoblastic leukemia (BCP-ALL) harbor fusions involving tyrosine kinase and cytokine receptors, such as *ABL1*, *PDGFRB*, *JAK2* and *CRLF2*, which are targeted by tyrosine kinase inhibitors (TKIs). In the present study, transcriptome analysis or multiplex reverse transcriptase–PCR analysis of 373 BCP-ALL patients without recurrent genetic abnormalities identified 29 patients with kinase fusions. Clinically, male predominance (male/female: 22/7), older age at onset (mean age at onset: 8.8 years) and a high white blood cell count at diagnosis (mean: 94 200/μl) reflected the predominance of National Cancer Institute high-risk (NCI-HR) patients (NCI-standard risk/HR: 8/21). Genetic analysis identified three patients with *ABL1* rearrangements, eight with *PDGFRB* rearrangements, two with *JAK2* rearrangements, three with *IgH-EPOR* and one with *NCOR1-LYN*. Of the 14 patients with *CRLF2* rearrangements, two harbored *IgH-EPOR* and *PDGFRB* rearrangements. *IKZF1* deletion was present in 16 of the 22 patients. The 5-year event-free and overall survival rates were 48.6±9.7% and 73.5±8.6%, respectively. The outcome was not satisfactory without sophisticated minimal residual disease-based stratification. Furthermore, the efficacy of TKIs combined with conventional chemotherapy without allogeneic hematopoietic stem cell transplantation in this cohort should be determined.

## Introduction

Modern therapeutic strategy of pediatric acute lymphoblastic leukemia (ALL), such as minimal residual disease-based risk-adopted treatment, progress of chemotherapy, supportive care and allogeneic hematopoietic stem cell transplantation (allo-HSCT) greatly improved the prognosis of pediatric ALL.^1^ However, approximately 15–20% patients experience relapse and show dismal prognosis.^1^ Additionally, late effect is also the considerable problem for the heavily treated patients, especially for the patients who received allo-HSCT. Thus it is quite important to treat high-risk ALL patients such as Ph+ ALL patients with less toxic chemotherapeutic regimen.^2^

Recent comprehensive genomic analyses revealed the genetic landscape of high-risk pediatric B-cell precursor ALL (BCP-ALL).^3, 4, 5, 6, 7^ In particular, a number of chimeric fusions, including those involving tyrosine kinase and cytokine receptors, were identified in a subgroup of BCP-ALL designated as Ph-/*BCR-ABL*-like ALL.^6, 7^ Because some of these genetic alterations may be treated by molecular targeted therapies, these patients may benefit from specific kinase inhibitor treatment.^8, 9^ However, a comprehensive analysis of the clinical characteristics of patients with these kinase fusions is necessary, despite recent reports providing clinical information on these patients.^7, 10^ The present study aimed to clarify the clinical characteristics of Philadelphia-negative BCP-ALL patients with kinase fusions in Japan by performing a genetic analysis of pediatric BCP-ALL to identify patients with kinase fusions. Clinical information was collected retrospectively and data were analyzed to identify prognostic factors in these patients.

## materials and Methods

### Patient cohort and samples

Diagnostic samples of bone marrow or peripheral blood were obtained from patients with pediatric BCP-ALL enrolled in and treated under clinical trial protocols of the Japan Association of Childhood Leukemia Study Group (JACLS)-ALL02 study (*n*=1252),^11, 12^ Tokyo Children's Cancer Study Group (TCCSG)-L04-16 (*n*=150), L06-16 (*n*=194), L07-16 (*n*=274) and L09-16 (*n*=607) studies,^13^ Childhood Cancer and Leukemia Study Group (CCLSG)-ALL2004 study (*n*=325)^14^ and Kyushu–Yamaguchi Childhood Cancer Study Group (KYCCSG)-ALL02 study (*n*=156).^15^ The diagnosis of BCP-ALL was based on morphological findings of bone marrow aspirates and immunophenotypic analyses of leukemic cells by flow cytometry. Conventional cytogenetic analyses and molecular studies were performed as part of the routine work-up in each protocol.^11, 12, 13, 14, 15^ Patients with recurrent fusion transcripts, including *ETV6-RUNX1*, *E2A-PBX1*, *MLL*-related fusion and *BCR-ABL*, and those with high hyperdiploidy were excluded that defined as B-others ALL. Down syndrome-related BCP-ALL was also excluded from this study. Informed consent was obtained from the guardians of patients according to the Declaration of Helsinki, and genetic study protocols were approved by the institutional review boards of the participating institutes.

### mRNA sequencing (mRNA-seq) and multiplex reverse transcriptase–PCR (mRT-PCR)

**Figure 1:**
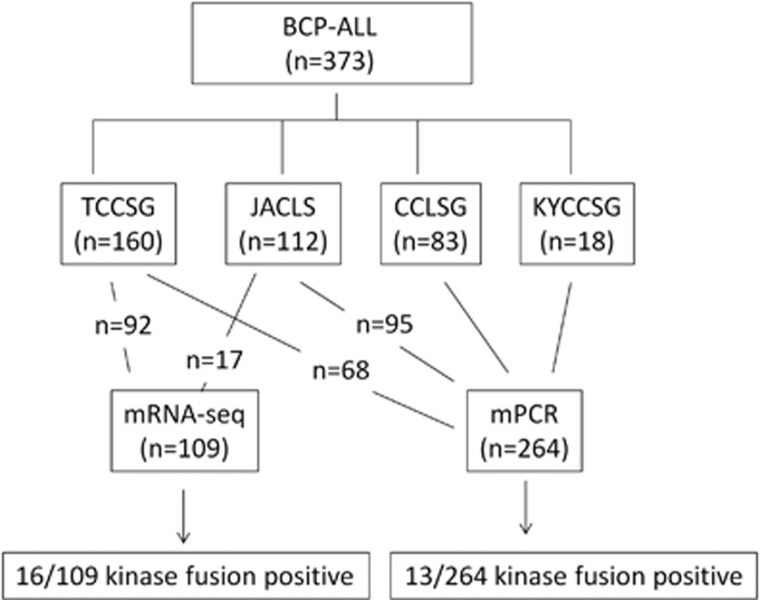
A consort diagram of genetic analysis of 373 patients. Samples were obtained from patients treated in the TCCSG (*n*=160), JACLS (*n*=112), CCLSG (*n*=83) and KYCCSG (*n*=18) cohorts. Ninety-two of the 160 in the TCCSG cohort and 17 of the 112 in the JACLS cohort were analyzed by mRNA-seq. The remaining 264 patients were analyzed by mRT-PCR. The kinase-activating fusions were identified in 16 of the 109 by mRNA-seq and in 13 of the 264 by mRT-PCR.

mRNA-seq or mRT-PCR analyses were performed in 373 BCP-ALL patients with sufficient RNA samples. mRNA-seq was performed in 92 patients in the TCCSG cohort and 17 patients in the JACLS cohort (total 109 patients) according to previously described methods.^16^ Briefly, the cDNA libraries were loaded on to the cBot (Illumina, Inc., San Diego, CA, USA) for clustering on a flow cell and then sequenced using a HiSeq1000 (Illumina). A paired-end run was performed using the SBS Kit (Illumina). Real-time analysis and basecalling was performed using the HiSeq Control Software Version 1.5 (Illumina). The chimeric transcripts were investigated by employing defuse and TopHat-Fusion, algorithms for gene fusion.^17^ All kinase fusions determined by mRNA-seq were validated by RT-PCR and Sanger sequencing. mRT-PCR was used in 264 patients (68 in the TCCSG cohort, 95 in the JACLS cohort, 83 in the CCLSG cohort and 18 in the KYCCSG cohort) to determine the presence of 15 kinase fusions, including *ZMIZ1-ABL1*, *SNX2-ABL1*, *FOXP1-ABL1*, *SFPO-ABL1*, *EML1-ABL1*, *NUP214-ABL1*, *RCSD1-ABL1*, *ETV6-ABL1*, *RANBP2-ABL1*, *STRN3-JAK2*, *BCR-JAK2*, *PAX5-JAK2*, *EBF1-PDGFRB*, *ATF7IP-PDGFRB* and *P2RY8-CRLF2*.^6, 16, 18^ The primers used in this study are listed in Supplementary Table S1.^6^ The consort diagram of the genetic analysis is described in Figure 1. The comparison of clinical characteristics of analyzed and non-analyzed patients in each cohort, such as TCCSG, JACLS, CCLSG and KYCCSG, are summarized in Supplementary Table S2. Although clinical characteristics were not significantly different between the analyzed and non-analyzed groups in TCCSG, CCLSG and KYCCSG cohorts, National Cancer Institute high-risk (NCI-HR) patients were more in the analyzed group in the JACLS cohort.

### Determination of *IKZF1* deletion and *JAK2* mutation

To identify the copy number abnormality of *IKZF1*, *PAX5*, *EBF1*, *ETV6*, *CDKN2A*, *CDKN2B*, *RB1* and *BTG1* in patients with kinase fusions, the SALSA Multiplex Ligation-dependent Probe Amplification (MLPA) Kit P335-A4 (MRC Holland, Amsterdam, The Netherlands) was used as described previously.^19^ Screening of *JAK2* exons 16, 20 and 21 (gene accession number NM 004972) mutations was performed in patients with *CRLF2* rearrangement, as described previously.^19^

### Gene set enrichment analysis

Gene expression profiles of the patients' samples analyzed by mRNA-seq were obtained as previously described.^16^ To assess similarity of gene expression profile between the kinase fusion-positive cases and the signature of *BCR-ABL1*-positive cases, gene set enrichment analysis was performed as previously described. ^16^

### Statistical analysis

Event-free survival (EFS) and overall survival (OS) rates were estimated using the Kaplan–Meier method, and differences were compared using the log-rank test. A *P*-value <0.05 (two-sided) was considered significant. EFS and OS were defined as the times from diagnosis to event (any death, relapse, secondary malignancy or failure of therapy) and from diagnosis to death from any cause or to the last follow-up, respectively. Patients without an event of interest were censored at the date of last contact. Hazard ratios for probability of relapse between subgroups were calculated using univariate Cox models. Other comparisons were performed using the χ^2^ test or Fisher exact test, as appropriate.

## Results

### Identification of kinase fusions in BCP-ALL

Twenty-nine patients with kinase fusions were identified (Table 1, Supplementary Table S3), of whom 16 were identified by mRNA-seq and 13 by mRT-PCR. All kinase fusions identified by mRNA-seq were confirmed to be in-frame with intact tyrosine kinase domain by RT-PCR and Sanger sequencing. The involved exons in each kinase fusion except *CRLF2*-related ones and *IgH-EPOR* are listed in Supplementary Table S4. It was likely that mRNA-seq was more sensitive to detect the kinase fusion (16 of the 109, 14.7% by mRNA-seq vs 13 of the 264, 4.9% by mRT-PCR), simply because only mRNA-seq can detect a novel kinase fusion. However, comparing the detection frequency of the 15 kinase fusions that were included in the mRT-PCR system, we identified 9 of the 109 (8.3%) by mRNA-seq and 13 of the 264 (4.9%) by mRT-PCR assay. Therefore, the sensitivity of two detection methods is not significantly different (9/109 vs 13/264, *P*=0.32).

**Figure 2:**
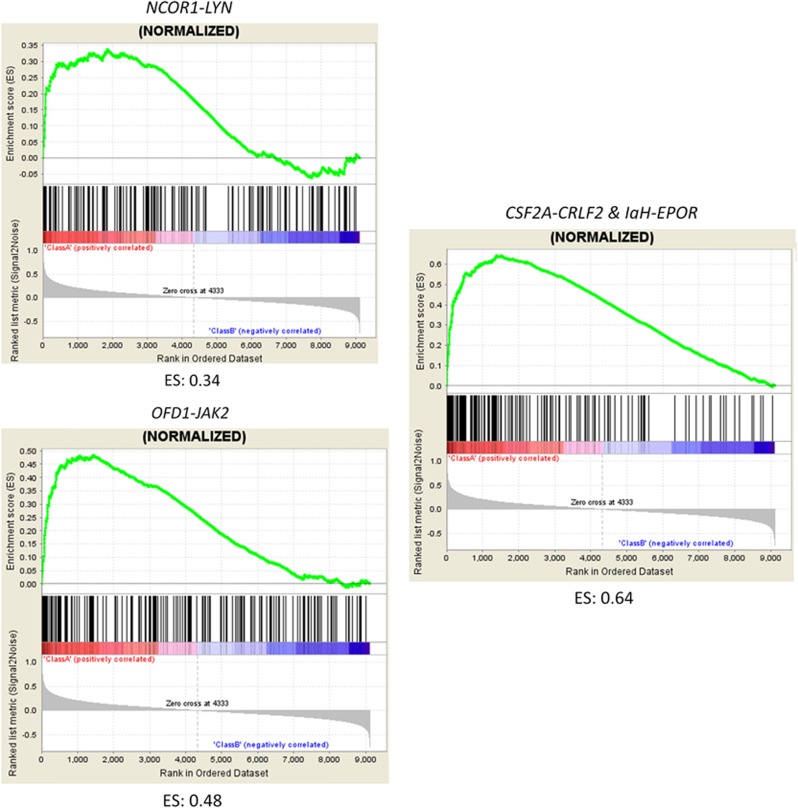
Gene set enrichment analysis plot of the patients with *NCOR1-LYN*, *OFD1-JAK2* and *CSF2RA-CRLF2*. The enrichment score (ES) is shown at the bottom of each graph. The positive ES means significant enrichment of the *BCR-ABL1* gene expression signature.

Regarding chimeric fusions, three patients had *ABL1* rearrangements (*SNX2*, *ZMIZ1* and *ETV6*), eight had *PDGFRB* rearrangements (*EBF1* in six patients and *ATF7IP*^18^ in two patients), two had *JAK2* rearrangements (*PAX5* and *OFD1*^20^), three had *IgH-EPOR* and one had *NCOR1-LYN*.^20^ Of the 14 patients with *CRLF2* rearrangements (*P2RY8* in 11 patients, *IgH* in 2 patients and *CSF2RA*^21^ in 1 patient), 2 harbored *IgH-EPOR* and *ATF7IP*-*PDGFRB* (Table 1). MLPA analysis detected *IKZF1* deletions in 16 of the 22 (72.7%) patients (Table 2, Supplementary Table S3). Mutational analysis of *JAK2* was performed in 12 of the 14 patients with *CRLF2* rearrangement and identified 2 of the 12 (16.7%) patients with *JAK2* R683-activating mutations (Table 2). The results of MLPA analysis in 29 kinase fusion-positive patients are summarized in Supplementary Table S3. Gene set enrichment analysis revealed that gene expression profile of the patients harboring kinase fusion except for *P2RY8-CRLF2* was similar to that of *BCR-ABL1*-positive patients (Figure 2).

### Clinical characteristics and outcomes of BCP-ALL patients with kinase fusions

Analysis of the clinical characteristics of the 29 patients showed male predominance (male/female: 22/7), older age at onset (median age at diagnosis: 8.8 years) and high white blood cell (WBC) count at diagnosis (median WBC count at diagnosis: 94 200/μl), which reflected the predominance of NCI-HR patients (NCI-standard risk (SR)/HR: 8/21) (Table 3). Thirteen of the 29 (44.8%) patients showed poor response to initial prednisolone (PSL) therapy. Ten patients (34.5%) who received allo-HSCT were in the first complete remission (CR), and six of them (60%) maintained first CR. Four patients (13.8%) did not respond to the initial treatment, and two of them were alive in CR by allo-HSCT. The remaining two patients died of treatment-related complications, including transplant-related mortality. Eleven patients (37.9%) experienced relapse after first CR, of whom six (54.5%) died; two of the six patients had received allo-HSCT after relapse. Only one patient who harbored *SNX2-ABL1* was treated with tyrosine kinase inhibitors, such as imatinib and dasatinib; the patient did not respond well to tyrosine kinase inhibitors in combination with chemotherapy at the first and second relapse.^16^ The clinical course of 29 patients is summarized in Supplementary Figure S1.

**Figure 3:**
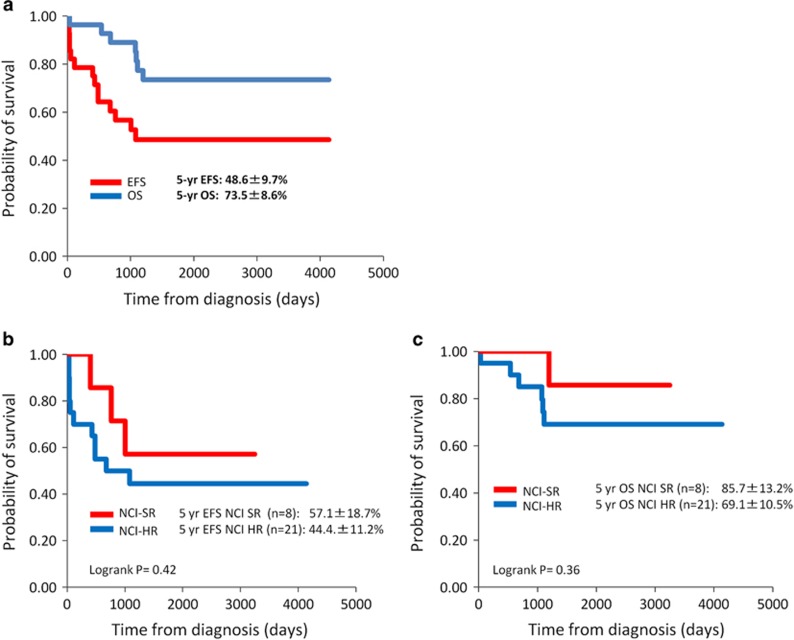
Probability of EFS and OS in 29 patients with kinase fusions (**a**) and according to NCI risk group. (**b**) EFS, (**c**) OS.

In the survival analysis, the 5-year EFS and OS rates were 48.6±9.7% and 73.5±8.6%, respectively, with a median follow-up period of 6.7 years (Figure 3a). To determine prognostic significance of kinase-activating fusions, we compared 5-year EFS between kinase fusion-positive (*n*=16) and -negative (*n*=93) in mRNA-seq cohort. The 5-year EFS rate was 41.0±13.0% in the kinase-activating fusion-positive group and 67.0±7.7% in the fusion-negative group, respectively. The prognosis of kinase-activating fusion-positive patients was significantly inferior to that of kinase fusion-negative patients (log-rank *P*=0.035). When mRNA-seq cohort was split into two groups based on the clinical trial, such as TCCSG (*n*=92) and JACLS cohorts (*n*=17), the 5-year EFS rate was 38.1±19.9% in activating kinase fusion-positive patients and 68.9±9.3% in activating kinase fusion-negative patients in the TCCSG cohort. The difference was statistically significant (log-rank *P*=0.039). On the other hand, the 5-year EFS was 50.0±20.4% in activating kinase fusion-positive B-others patients and 54.6±15.0% in negative B-others patients in the JACLS cohort. The difference was not statistically significant (log-rank *P*=0.89). Because all of the 17 patients in the JACLS cohort have *IKZF1* deletion, the prognosis of these patients was poor irrespective of the presence of kinase-activating fusions. According to the NCI risk classification, the 5-year EFS rate was 57.1±18.7% in the SR group and 44.4±11.2% in the HR group. The 5-year OS rate was 85.7±13.2% in the SR group and 65.8±10.5% in the HR group (Figures 3b and c). Although univariate analysis was performed to determine the factors related to inferior EFS or OS in 29 patients, none of the covariates such as age at diagnosis, WBC count at diagnosis, NCI risk, initial PSL response, *IKZF1* deletion or allo-HSCT in first CR were statistically significant (Table 4).

According to the type of chimeric fusions, among patients with *ABL*-class rearrangements, such as *ABL1*, *PDGFRB* and *LYN*, which could be targets of imatinib or dasatinib, the 5-year EFS and OS rates were 63.6±14.5% and 90.0±9.5%, respectively (Supplementary Figures S2A and B). Among patients with *JAK*-class rearrangements, such as *JAK2*, *CRLF2* and *EPOR*, which could be targets of JAK2 inhibitors, the 5-year EFS and OS rates were 37.5±12.1% and 62.5±12.1%, respectively (Supplementary Figures S2A and B). There were no significant differences in EFS and OS between the two genetic subgroups (log-rank *P*=0.42 and 0.18, respectively). No significantly different factors, such as age at onset, initial WBC count, NCI risk, *IKZF1* deletion, initial PSL response and the number of patients receiving allo-HSCT in first CR, were observed between the two genetic subgroups (Table 5).

## Discussion

Roberts *et al.*^7^ identified kinase fusions in 172 of the 264 patients with Ph-like ALL using mRNA-seq. Although 106 (61.6%) of the 172 patients harbored *CRLF2*-related fusions (45 *P2RY8-CRLF2* and 61 *IgH-CRLF2*), the chimeric fusions identified in the remaining 66 patients varied. In detail, 36 (20.9%) patients harbored *ABL*-class rearrangements, including *ABL1*, *ABL2*, *CSF1R* and *PDGFRB*; 28 (16.3%) patients harbored *EPOR* or *JAK2* rearrangement; and the remaining 2 (1.2%) patients harbored rare kinase fusions, such as *ETV6-NTRK3* and *ZFAND3-DGKH*. *IKZF1* deletion was present in 45 (68.2%) of the 66 patients with kinase fusions, excluding *CRLF2*-related fusions. In addition, 68 (55%) of the 106 patients with *CRLF2* rearrangement harbored *JAK2*-activating mutation.

In the present study, we analyzed 373 patients with BCP-ALL without hyperdiploid karyotype and recurrent fusions, such as *ETV6-RUNX1*, *E2A-PBX1*, *MLL*-related fusions and *BCR-ABL*, because we did not have enough data for gene expression profiling, which is mandatory for the diagnosis of Ph-like ALL. We identified 29 kinase fusion-positive patients: 14 patients with *CRLF2* rearrangements, 12 patients with *ABL*-class rearrangements, and 5 patients with *EPOR* or *JAK2* rearrangement. Our retrospective analysis confirmed three types of kinase rearrangements, including *CRLF2*, *ABL1*-class and *JAK2*/*EPOR*, which were dominant, although we may have missed several patients with kinase fusions owing to lack of data in 264 patients who were not analyzed by mRNA-seq. A high prevalence (16 of the 22, 72.7%) of *IKZF1* deletion in these patients was also confirmed in our study, suggesting that kinase fusion-positive BCP-ALL patients are biologically similar to Ph+ ALL patients.^22^ However, *JAK2* mutation was present in only 2 (16.7%) of the 12 patients with *CRLF2* rearrangements, which was a lower rate than that (55%) reported in previous studies.^5^ The reason for this discrepancy is unclear. Because other genetic alterations related to the JAK-STAT pathway, such as activating mutations in the *IL7R*, *FLT3*, *JAK1* and *JAK3* genes and the deletion of *SH2B3*, were not investigated in our study, these genetic alterations may have replaced *JAK2*-activating mutations in our cohort.^6, 7^ Additional comprehensive genetic studies are warranted to identify the therapeutic targets in our cohort.

We confirmed that the clinical characteristics of patients with kinase fusions are those commonly associated with Ph+ and Ph-like ALL, such as older age at diagnosis, male predominance and high WBC count at diagnosis.^6, 7, 23, 24^ In terms of treatment response, we showed that the prevalence of PSL poor responders was high in this subgroup, compared with the findings of the BFM2000 study (44.8% vs 6.3%, *P*<0.01).^25^ Compared with the kinase-activating fusion-negative patients in this study, the rate of induction failure was also high in this subgroup (12.9% vs 2.1%, *P*=0.004). However, the PSL poor responders and induction failure rates in our study did not differ significantly from those reported in the Ph+ ALL cohort (44.8% vs 21.4%, *P*=0.07 and 13.8% vs 14%, *P*=0.86, respectively).^24^ These findings suggested clinical similarities between Ph+ ALL and BCP-ALL with kinase fusions other than *BCR-ABL*.

The prognostic impact of Ph-like signature is controversial.^7, 10, 23, 26^ In addition, prognostic significance of kinase-activating fusions should be determined. In the present study, the 5-year EFS rates of kinase fusion-positive patients was significantly inferior to those of kinase fusion-negative patients in the mRNA-seq cohort, which included non-biased B-others (log-rank *P*=0.035). The limitation of this study was that mRNA-seq was performed only in 109 of the 373 (29.2%) patients. Complete genetic study of larger cohort is needed to clarify the prognostic significance of kinase-activating fusions in pediatric BCP-ALL.

Although information about risk factors associated with Ph-like ALL is limited, Roberts *et al.*^7, 10^ suggested that age at onset and minimal residual disease status are related to poor prognosis. In the present study, the small sample size prevented identification of risk factors related to inferior EFS and OS in our cohort, such as age at diagnosis, NCI risk and the presence of *IKZF1* deletion. Furthermore, we focused on analyzing differences in the biological or clinical characteristics between genetic subgroups, such as those with *ABL*-class rearrangements and *JAK*-class rearrangements. Although EFS and OS tend to be low among patients with *JAK*-class rearrangements, there were no statistically significant differences between groups (data not shown). Furthermore, we could not find any difference in clinical and biological features between the two genetic subgroups (Table 5). However, our study was limited by its small sample size, its retrospective nature, the heterogeneity of treatment protocols and no data of minimal residual disease status. Thus further studies using larger cohorts are required to determine the prognostic factors in this unique subgroup. International collaborating studies are warranted in the near future.

Finally, the rapid identification of tyrosine kinase inhibitor-eligible patients is mandatory. The establishment of systematic screening systems is currently in progress in the pediatric study groups. For example, the Children's Oncology Group used a low-density gene expression array to identify Ph-like ALL.^7^ We are currently developing fluorescence *in situ* hybridization protocols for the detection of *ABL1*, *PDGFRB* and *JAK2* rearrangements, which are targetable and relatively frequent genetic abnormalities. In the future, efficient screening systems for the identification of rare but treatable kinase fusions should be established to enable the design of tailored therapy protocols for patients with high-risk BCP-ALL.

## Figures and Tables

**Table 1:** Kinase fusions identified in this study

*Kinase gene*	*Fusion partners (*n*)*	*Patients (*n*)*	*5′ Genes*
*ABL1*	3	3	*SNX1*, *ZIMZ1*, *ETV6*
*PDGFRB*	2	8^a^	*EBF1*, *ATF7IP*
*CRLF2*	3	14^a^	*P2RY8*, *IgH*, *CSF2RA*
*JAK2*	2	2	*PAX5*, *OFD1*
*EPOR*	1	3^a^	*IgH*
*LYN*	1	1	*NCOR1*

a There were two patients who harbored two kinase fusions. One had ATF7IP-PDGFRB and P2RY8-CRLF2, and another patient had IgH-EPOR and CSF2RA-CRLF2.

**Table 2:** *IKZF1* and *JAK2* status of kinase fusion-positive patients in this study

*Kinase fusion*	*IKZF1*			*JAK2*		
	*WT*	*Deletion*	*ND*	*WT*	*Mutation*	*ND*
*P2RY8-CRLF2*	6	3	1	8	1^a^	1
*IgH-CRLF2*	0	1	1	0	1^b^	1
*CSF2RA-CRLF2+IgH-EPOR*	0	1	0	1	0	0
*EBF1-PDGFRB*	0	5	1	—	—	—
*ATF7IP-PDGFRB+P2RY8-CRLF2*	0	1	0	1	0	0
*ATF7IP-PDGFRB*	0	0	1	—	—	—
*PAX5-JAK2*	0	1	0	—	—	—
*OFD1-JAK2*	0	1	0	1	0	0
*IgH-EPOR*	0	1	1	—	—	—
*SNX2-ABL1*	0	0	1	—	—	—
*ZMIZI1-ABL1*	0	0	1	—	—	—
*ETV6-ABL1*	0	1	0	—	—	—
*NCOR1-LYN*	0	1	0	1	0	0

Abbreviations: ND, not determined; WT, wild type.

a R683S mutation was identified.

b Both R683S and R683G mutations were identified in this patient.

**Table 3:** Clinical characteristics of 29 patients with kinase fusions

Age (years)	
Median	8.8
Range	1.9–16
	
*WBC count*	
Median (× 10^3^/μl)	94.2
Range	0.6–420
	
*Sex*	
Male	22
Female	7
	
*NCI*	
SR	8
HR	21
	
*PSL response*	
PGR	16
PPR	13
	
*Induction failure*	
Yes	4
No	25
	
*Allo-HSCT in first CR*	
Yes	10
No	19
	
*Outcome*	
Alive	21
Dead	8

Abbreviations: allo-HSCT, allogeneic hematopoietic stem cell transplantation; CR, complete remission; HR, high risk; NCI, National Cancer Institute; PGR, prednisolone good responder; PPR, prednisolone poor responder; PSL, prednisolone; SR, standard risk; WBC, white blood cell.

**Table 4:** Univariate Cox model of event-free and overall survival of the analyzed patients

*Variable*	*Hazard ratio*	P	*95% CI*	*Variable*	*Hazard ratio*	P	*95% CI*
	*Event-free survival*				*Overall survival*					
Age (years) at diagnosis (10−18 vs 1–9)	1.93	0.20	0.698–5.360	Age (years) at diagnosis (10−18 vs 1–9)	1.83	0.37	0.489–6.808			
WBC (/μl) at diagnosis (>50 000 vs <50 000)	1.33	0.30	0.777–2.276	WBC (/μl) (>50 000 vs <50 000)	1.60	0.25	0.717–3.560			
NCI risk (HR vs SR)	2.28	0.20	0.643–8.110	NCI risk (HR vs SR)	3.90	0.20	0.478–31.73			
* IKZF1* status (deletion vs WT)	3.61	0.10	0.776–16.80	* IKZF1* status (deletion vs WT)	2.57	0.40	0.287–23.02			
PSL response (PPR vs PGR)	1.73	0.29	0.624–4.80	PSL response (PPR vs PGR)	0.79	0.75	0.188–3.307			
Allo-HSCT in first CR (yes vs no)	1.19	0.80	0.321–4.394	Allo-HSCT in first CR (yes vs no)	0.22	0.15	0.027–1.723			

Abbreviations: allo-HSCT, allogeneic hematopoietic stem cell transplantation; CI, confidence interval; CR, complete remission; HR, high risk; NCI, National Cancer Institute; PGR, prednisolone good responder; PPR, prednisolone poor responder; PSL, prednisolone; SR, standard risk; WBC, white blood cell; WT, wild type.

**Table 5:** Comparison of the characteristics between two genetic subgroups

	*PDGFRB+ABL1+LYN*	*JAK2+EPOR+CRLF2*	P
*Age (years) at diagnosis*			
<10	6	10	0.92
>10	6	7	
			
*WBC (/μl) at diagnosis*			
<50000	3	8	0.27
>50000	9	9	
			
*NCI risk*			
SR	2	6	0.41
HR	10	11	
			
*IKZF1 status*			
WT	1	7	0.18
del	7	8	
			
*PSL response*			
PGR	5	11	0.5
PPR	7	7	
			
*Relapse or IF*			
+	5	10	0.59
−	7	7	
			
*Survival*			
Alive	10	11	0.41
Dead	2	6	
			
*Allo-HSCT in first CR*			
+	5	5	0.69
−	7	12	

Abbreviations: allo-HSCT, allogeneic hematopoietic stem cell transplantation; CR, complete remission; HR, high risk; IF, induction failure; NCI, National Cancer Institute; PGR, prednisolone good responder; PPR, prednisolone poor responder; PSL, prednisolone; SR, standard risk; WBC, white blood cell; WT, wild type.
