# Supplementary figures and images for: Characterization of pediatric Philadelphia-negative B-cell precursor acute lymphoblastic leukemia with kinase fusions in Japan
Source: Blood Cancer J. 2016 May 13;6(5):e419–. doi: 10.1038/bcj.2016.28 (PMC4916297; doi:10.1038/bcj.2016.28)

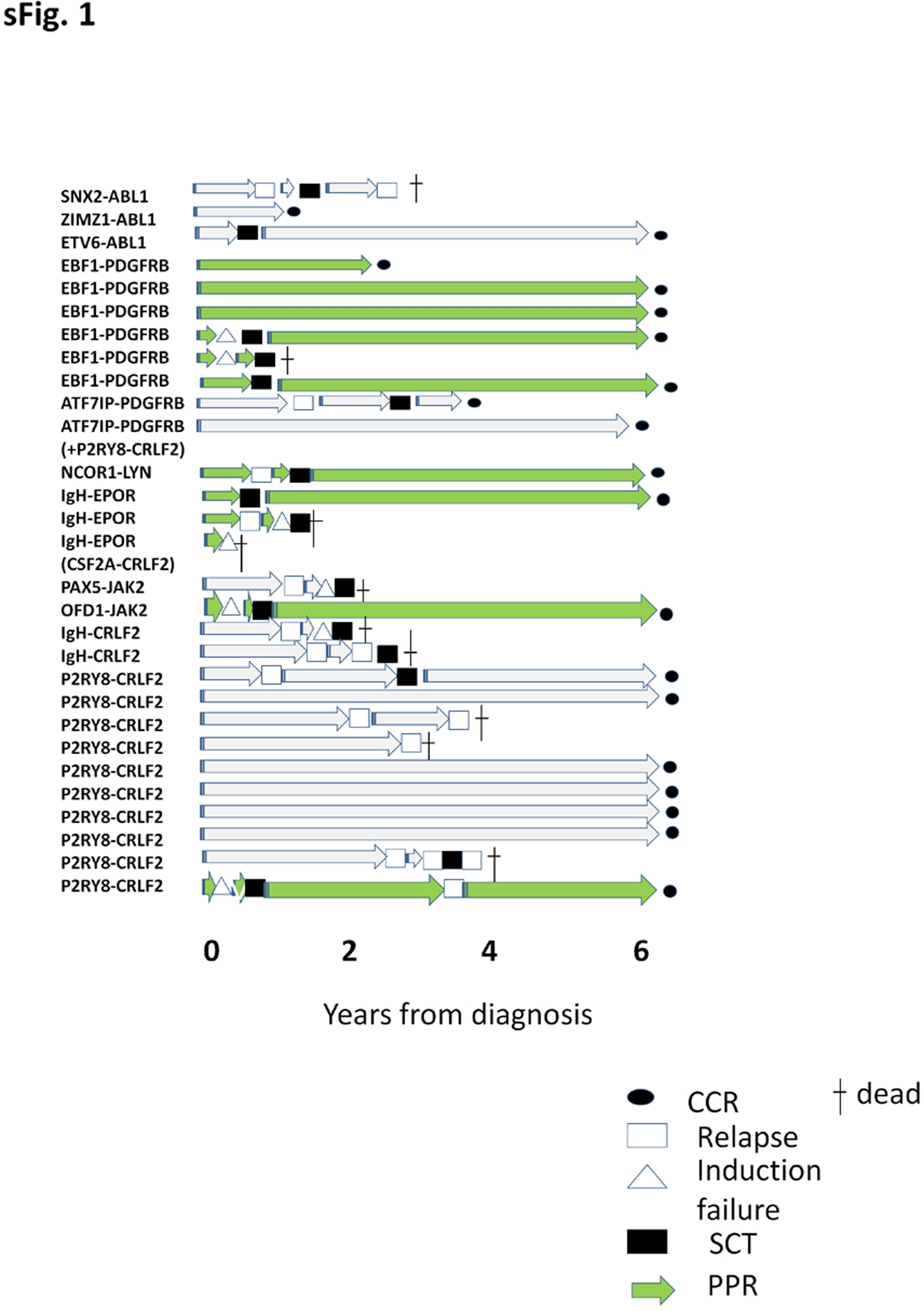

Supplement: Supplementary Figure 1 [file bcj201628x1.tif]

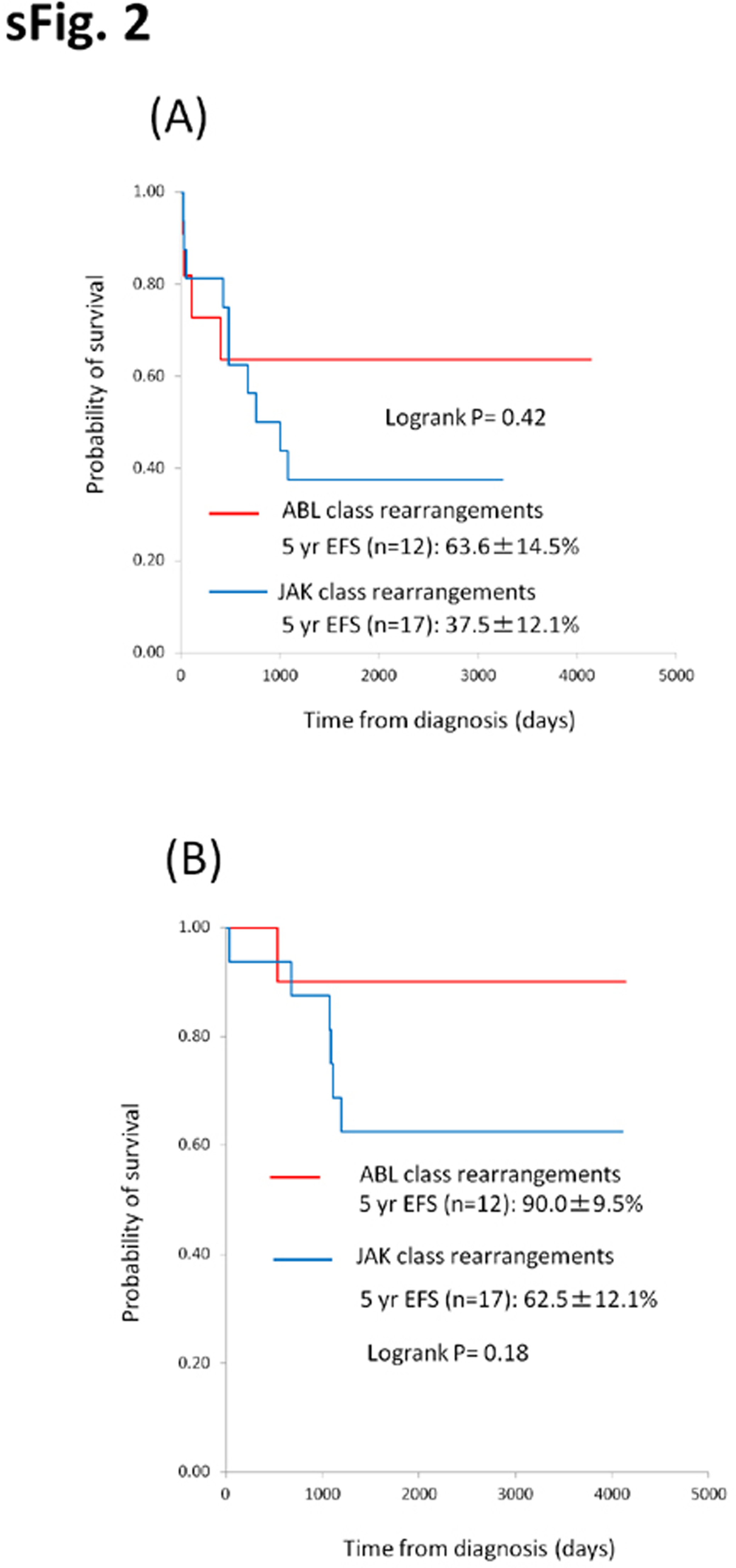

Supplement: Supplementary Figure 2 [file bcj201628x2.tif]
