# Supplementary material for: Characterization of pediatric Philadelphia-negative B-cell precursor acute lymphoblastic leukemia with kinase fusions in Japan
Source: Blood Cancer J. 2016 May 13;6(5):e419–. doi: 10.1038/bcj.2016.28 (PMC4916297; doi:10.1038/bcj.2016.28)
Supplement: Supplementary Tables [file bcj201628x3.docx]

**Supplementary Table 1.** Primer pairs used for multiplex PCR in this study

| Fusions | Forward | Reverse |
| --- | --- | --- |
| *1^st^ P2RY8-CRLF2* | 5'- CACGAACACCTTCTCAAGCA -3' | 5'- GTCCCATTCCTGATGGAGAAA -3' |
| *2^nd^ P2RY8-CRLF2* | 5'- TGTTACCTGGAGACCCTCTG -3' | 5'- GTTGAATCTGCTCCTCCTTG -3' |
| *SNX2-ABL1* | 5'- AAGAGTATGTCTGCTCCCGTGATCTT -3' | 5'- CCACCGTTGAATGATGATGAACC-3' |
| *ZMIZI-ABL1* | 5'- TACCCTGGGGGTCCTAACG -3' | 5'- CCACCGTTGAATGATGATGAACC-3' |
|  | 5'- AGTATTACAAGCCAGAACAGTTTAATGGA -3' | 5'- AGTTCCAACGAGCGGCTTCACTCAGA -3' |
| *FOXP1-ABL1* | 5'- GCAGTATGGACAGTGGATGAAGTA -3' | 5'- CCACCGTTGAATGATGATGAACC-3' |
| *SFPQ-ABL1* | 5'- GATGCCTATCATGAACATCAGGC -3' | 5'- CCACCGTTGAATGATGATGAACC-3' |
| *EML1-ABL1* | 5'- CACTCACTGGGAGGTGGTTT -3' | 5'- CCACCGTTGAATGATGATGAACC-3' |
| *ETV6-ABL1* | 5'- AAATCACCGGCCTTCTCCTG -3' | 5'- CCACCGTTGAATGATGATGAACC-3' |
|  | 5'- CGGCACTCCGTGGATTTCAAACAGT -3' | 5'- TGTAGTTGCTTGGGACCCAGCCTTG -3' |
| *RCSD1-ABL1* | 5'- GGACAGCGGGGATCGTGAG -3' | 5'- CCACCGTTGAATGATGATGAACC-3' |
|  | 5'- CAGCCAGTAAACCAACCCGAAGGAA -3' | 5'- GCTTGTTGCGCTTTGGGGCTGGA -3' |
| *NUP214-ABL1* | 5'- CAGTGGCCTTGGAGGAAAACCCAGT -3' | 5'- TGTAGTTGCTTGGGACCCAGCCTTG -3' |
| *RANBP2-ABL1* | 5'- TGGTTCTTTGCGAAATGCAGATTCA -3' | 5'- GCCATTTTTGGTTTGGGCTTCACAC -3' |
| *EBF1-PDGFRβ* | 5'- CACGAGCATGAACGGTACGGCTCT-3' | 5'- TTTCATCGTGGCCTGAGAATGGCTC -3' |
|  | 5'- GGGCGTGAATTCGTTCAGTG -3' | 5'- ATCGGATCTCGTAACGTGGC -3' |
| *ATF7IP- PDGFRβ* | 5'- CGTGAATGTAACACATCGTCCA -3' | 5'- AGCTCCCACGTGGAGTCATAG -3' |
| *BCR-JAK2* | 5'- GTGCCATAAGCGGCACCGGCACT -3' | 5'- TCTCCTCCACTGCAGATTTCCCACA -3' |
| *STRN3-JAK2* | 5'- ATGATGAGCTGCCCCACATCCCTTC -3' | 5'- CGGCACATCTCCACACTCCCAAAAT -3' |
| *PAX5-JAK2* | 5'- ACCAACCAGTCCCAGCTTCCAGCTTCCAGTCA -3' | 5'- CGGCACATCTCCACACTCCCAAAAT -3 |

**Supplementary Table 2.** Comparison of clinical characteristics of patients depending on whether they were included in the genetic analyses in each cohort .

(A) TCCSG cohort

|  | TCCSG | | *P* |
| --- | --- | --- | --- |
|  | Analyzed | Non-analyzed |  |
| Gender (Male/Female) | 76/77 | 247/181 | 0.09 |
| Age (yrs) at diagnosis  median (range) | 5 (1–17) | 4 (0–17) | 0.1 |
|  |  |  |  |
| WBC count, (cells/μl) median (range) | 8100  (700–788000) | 8100  (800–597000) | 0.57 |
| NCI risk group, SR/HR | 89/50 | 262/115 | 0.24 |
| Observation period (days),  median (range) | 1145.5 (0–2982) | 939.5 (0–2978) | 0.07 |
| Outcome |  |  | 0.33 |
| alive | 125 | 349 |  |
| dead | 12 | 23 |  |

TCCSG, Tokyo Children’s Cancer Study Group; WBC, white blood cell; NCI, National Cancer Institute; SR, standard risk; HR, high risk.

(B) JACLS cohort

|  | JACLS | | *P* |
| --- | --- | --- | --- |
|  | Analyzed | Non-analyzed |  |
| Gender (Male/Female) | 64/50 | 259/232 | 0.51 |
| Age (yrs) at diagnosis  median (range) | 7 (1-18) | 5 (1-17) | 0.01 |
|  |  |  |  |
| WBC count, (cells/μl)  median (range) | 17,900 (600-420,000) | 5,800  (430-328,900) | <0.01 |
| NCI risk group, SR/HR | 55/59 | 343/148 | <0.01 |
| Observation period (days),  median (range) | 2,136 (26-4,284) | 2,285 (13-4,103) | 0.15 |
| Outcome |  |  | 0.05 |
| alive | 94 | 437 |  |
| dead | 20 | 54 |  |

JACLS, Japan Association of Childhood Leukemia Study Group; WBC, white blood cell; NCI, National Cancer Institute; SR, standard risk; HR, high risk.

(C) CCLSG cohort

|  | CCLSG | | *P* |
| --- | --- | --- | --- |
|  | Analyzed | Non-analyzed |  |
| Gender (Male/Female) | 50/32 | 140/103 | 0.607 |
| Age (yrs) at diagnosis  median (range) | 5 (1-18) | 5 (1-18) | 0.428 |
|  |  |  |  |
| WBC count, (cells/μl)  median (range) | 9,635 (900-192,700) | 10,050 (770-747,500) | 0.277 |
| NCI risk group, SR/HR | 52/30 | 155/88 | 1.000 |
| Observation period (days)  median (range) | 3,307 (347-3,941) | 2,902 (29-4,042) | 0.006 |
| Outcome |  |  |  |
| alive | 75 | 217 | 0.58 |
| dead | 7 | 26 |  |

CCLSG, Childhood Cancer and Leukemia Study Group; WBC, white blood cell; NCI, National Cancer Institute; SR, standard risk; HR, high risk.

(D) KYCCSG cohort

|  | KYCCSG | | *P* |
| --- | --- | --- | --- |
|  | Analyzed | Non-analyzed |  |
| Gender (Male/Female) | 9/9 | 12/9 | 0.75 |
| Age (yrs) at diagnosis  median (range), | 6 (2-14) | 6 (1-16) | 0.71 |
|  |  |  |  |
| WBC count, cells/μl  median (range) | 22820 (1800-1195500) | 6250 (2100－260040) | 0.27 |
| NCI risk group, SR/HR | 12/6 | 12/9 | 0.74 |
| Observation period,  median  (range) | 2430  (120-3236) | 2531  (79-3361) | 0.96 |
| Outcome |  |  |  |
| alive | 16 | 18 | 0.65 |
| dead | 2 | 3 |  |

KYCCSG, Kyushu-Yamaguchi Childhood Cancer Study Group **;** WBC, white blood cell; NCI, National Cancer Institute; SR, standard risk; HR, high risk.

**Supplementary Table 3.** 29 patients’ characteristics, detection method of kinase fusion and summary of MLPA analysis

|  | age at onset (yrs) | Sex | Initial WBC count (/ μl) | Kinase fusion | detection method | Results of MLPA |
| --- | --- | --- | --- | --- | --- | --- |
| 1 | 9 | M | 5800 | *P2RY8-CRLF2* | mRT-PCR | *IKZF1, BTG1, PAX5, CDKN2B* deletion |
| 2 | 4 | F | 600 | *P2RY8-CRLF2* | mRT-PCR | *BTG1, PAX5, CDKN2A/2B* deletion |
| 3 | 7 | M | 2300 | *P2RY8-CRLF2* | mRT-PCR | *BTG1, PAX5, CDKN2B* deletion |
| 4 | 2 | M | 6590 | *P2RY8-CRLF2* | mRT-PCR | No deletion |
| 5 | 5 | M | 4300 | *P2RY8-CRLF2* | mRT-PCR | *PAX5* |
| 6 | 11 | M | 10800 | *P2RY8-CRLF2* | mRNA-seq | *IKZF1, BTG1, PAX5, CDKN2A/2B* deletion |
| 7 | 11 | F | 10900 | *P2RY8-CRLF2* | mRT-PCR | *CDKN2A/2B* deletion |
| 8 | 3 | M | 35200 | *P2RY8-CRLF2* | mRNA-seq | *PAX5, CDKN2A/2B* deletion |
| 9 | 2 | M | 94200 | *P2RY8-CRLF2* | mRT-PCR | *IKZF1, CDKN2A/2B* deletion |
| 10 | 1 | M | 109700 | *P2RY8-CRLF2* | mRT-PCR | ND |
| 11 | 8 | M | 374800 | *IgH-CRLF2* | mRNA-seq | *IKZF1, EBF1, CDKN2A/2B* deletion |
| 12 | 13 | F | 165400 | *IgH-CRLF2* | mRNA-seq | ND |
| 13 | 15 | M | 337500 | *CSF2RA-CRLF2*  *IgH-EPOR* | mRNA-seq | *IKZF1* deletion |
| 14 | 10 | M | 420000 | *EBF1-PDGFRB* | mRNA-seq | *IKZF1, EBF1, PAX5, CDKN2A/2B* deletion |
| 15 | 12 | M | 358900 | *EBF1-PDGFRB* | mRNA-seq | *IKZF1, PAX5, EBF1* deletion |
| 16 | 16 | F | 162000 | *EBF1-PDGFRB* | mPCR | *IKZF1, EBF1, PAX5, CDKN2A/2B* deletion |
| 17 | 15 | M | 31400 | *EBF1-PDGFRB* | mRNA-seq | ND |
| 18 | 12 | M | 162500 | *EBF1-PDGFRB* | mRNA-seq | *IKZF1, PAX5* deletion |
| 19 | 12 | M | 147400 | *EBF1-PDGFRB* | mPCR | *IKZF1* deletion |
| 20 | 4 | M | 50100 | *ATF7IP-PDGFRB*  *P2RY8-CRLF2* | mRNA-seq | *IKZF1, PAX5, CDKN2A/2B* deletion |
| 21 | 8 | M | 4900 | *ATF7IP-PDGFRB* | mPCR | ND |
| 22 | 10 | F | 100900 | *PAX5-JAK2* | mPCR | *IKZF1* deletion |
| 23 | 3 | M | 185800 | *OFD1-JAK2* | mRNA-seq | *IKZF1, EBF1, PAX5, CDKN2A* deletion |
| 24 | 15 | F | 86800 | *IgH-EPOR* | mRNA-seq | *IKZF1, PAX5* deletion |
| 25 | 11 | M | 54900 | *IgH-EPOR* | mRNA-seq | ND |
| 26 | 7 | M | 115400 | *SNX2-ABL1* | mRNA-seq | ND |
| 27 | 5 | M | 15200 | *ZMIZI1-ABL1* | mRNA-seq | ND |
| 28 | 3 | M | 109600 | *ETV6-ABL1* | mPCR | *IKZF1, CDKN2A* deletion |
| 29 | 8 | F | 293000 | *NCOR1-LYN* | mRNA-seq | *IKZF1, CDKN2A/2B, BTG1* deletion |

WBC, white blood cell; MLPA, multiplex ligation-dependent probe amplification; M, male; F, female; mRT-PCR, multiplex RT-PCR; mRNA-seq, messenger RNA sequencing; ND, not done.

**Supplementary Table 4.** The list of involved exons in each kinase fusions in this study

| Kinase fusion | Involved exons in fusion point | |
| --- | --- | --- |
|  | 5’ | 3’ |
| *EBF1-PDGFRB* | *EBF1* exon 14 | *PDGFRB* exon 11 |
| *ATF7IP-PDGFRB* | *ATF7IP* exon 13 | *PDGFRB* exon 11 |
| *PAX5-JAK2* | *PAX5* exon | *JAK2* exon |
| *OFD1-JAK2* | *OFD1* exon 22 | *JAK2* exon 13 |
| *SNX2-ABL1* | *SNX2* exon 3 | *ABL1* exon 4 |
| *ZMIZ1-ABL1* | *ZMIZ1* exon | *ABL1* exon |
| *ETV6-ABL1* | *ETV6* exon | *ABL1* exon |
| *NCOR1-LYN* | *NCOR1* exon 34 | *LYN* exon 7 |
