# Supplementary material for: Characterization of pediatric Philadelphia-negative B-cell precursor acute lymphoblastic leukemia with kinase fusions in Japan
Source: Blood Cancer J. 2016 May 13;6(5):e419–. doi: 10.1038/bcj.2016.28 (PMC4916297; doi:10.1038/bcj.2016.28)
Supplement: Supplementary Information [file bcj201628x4.docx]

**Supplementary Figure 1.**

Summary of clinical course of 29 patients.

Black circle indicates continuous complete remission. White square indicates relapse. White triangle indicates induction failure. Black square indicates stem cell transplantation (SCT). Green line indicates prednisolone poor response (PPR).

**Supplementary Figure 2**

Probability of event-free survival (EFS) and overall survival (OS) in patients with kinase fusions according to genetic subgroup. (A) EFS, (B) OS.
